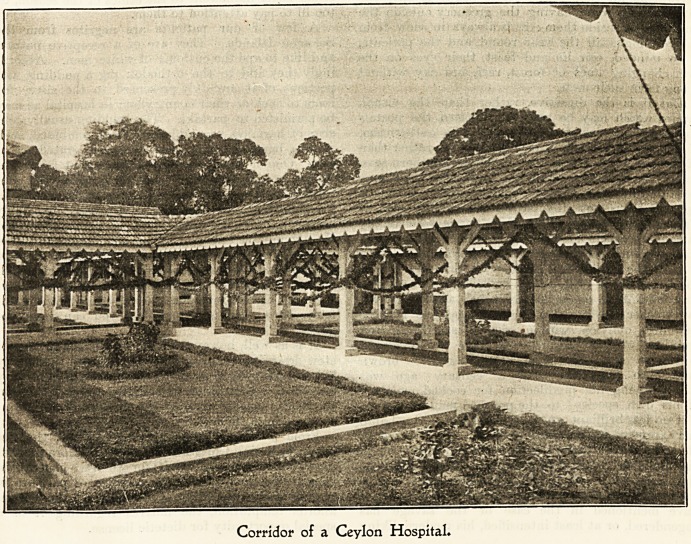# Our Christmas Supplement

**Published:** 1901-12-14

**Authors:** 


### OUR CHRISTMAS SUPPLEMENT.

### Hospitals within the Empire.

A LINK CONNECTING MANY LANDS.

The interest that has been aroused in all things pertaining to our colonies by the visit of the Heir Apparent and his wife to these distant lands leads us to think that we may enlist support for hospitals at home by showing in our Christmas supplement this year how the great festival of Christmas is observed in hospitals beyond the seas, how in every clime the seed sown by our English hospitals is bearing good fruit, how in every colony replicas of our English hospitals have been established—like them even to their Christmas festivals—and how the English hospital system has become one of the links of our great Empire. The following sketches which have been written on the spot by matrons, sisters, and staff nurses—and the accompanying illustrations—do far more than merely describe the efforts that are made in the most remote parts of the realms over which the King has sway to brighten the lot of suffering humanity at a time when all the world keeps high holiday; they also afford remarkable evidence of the continuous development of the hospital movement which had its origin in the Mother Country, and demonstrate afresh the fact that the methods, the treatment, and the spirit which prevail in the charitable institutions of Great Britain, are successfully and enthusiastically emulated in those of Greater Britain.

Nothing, we think, will strike those who read the contributions from our correspondents—which describe how Christmas was celebrated last year—more than the points of resemblance between the mode of its celebration in a great London hospital, and the mode in which it is kept in a hospital in Australia, Canada, South Africa, or the South Sea Islands. Of course there are points of difference. A Christmas tree in the open, under the stars, as in New South Wales, would not be possible in England. The extreme heat and the scent of roses in the air, which are characteristics of Christmas in South Africa, neither fatigue, nor gratify, nurses here. There is room for doubt as to the wisdom of using Chinese lanterns anywhere for decorative purposes, but the reduction of the candles by the heat of the sun to miniature copies of the Tower of Pisa, which troubled the mind of a Hong Kong nurse, is not a contingency that need occasion anxiety at Guy's. Nor does pork, the ideal Christmas dish of the patients in a Fiji hospital, figure on the bill of fare at King's. But even in colonies where the temperature in the shade on Christmas Day is 80°, there is a blazing sun, and holly is unobtainable, the hospital wards are gaily decorated; entertainments only slightly differing from those at home are provided for the benefit of the patients and participated in by visitors; the Christmas tree is a centre of interest, and presents are freely distributed. A similar state of things prevails in the representative institutions which do so much to convince the dusky natives of India that England means well towards them, and in the Punjab no pains are spared to save the inmates of the hospitals from having their sufferings aggravated by home-sickness. Throughout Australia the old English custom of roast beef and plum pudding for dinner is followed, and even in a hospital in the centre of the goldfields the old home plan of sisters and nurses accompanying Santa Claus in the distribution of his gifts on Christmas Eve is faithfully carried out. In Canada, where it is as easy to make the wards look like Christmas as it is in Great Ormond Street, the programme of the season still more closely resembles that of the home institutions, with the variation in one of the Toronto hospitals of hymn-singing by choir boys instead of comic songs by medical students; while a glance at the manner in which the festival is honoured in Capetown and Pretoria indicates that British soldiers wounded on the field of battle or Colonials stricken with disease, are made to feel as much as possible in touch with the Christmas rejoicings "at home." Our illustrations tell the same story. The exterior of the handsome building in Camden might be that of one of the principal English hospitals, except that we have fewer acres to spare here than they have in New South Wales. The pictures of the interior of wards in Australia might well be those of wards at home—wards with all modern appliances and comforts, lofty and admirably arranged. Even the pretty corridor of a Ceylon hospital is a sign of progress, and the group of nurses on the lawn at Suva shows that the need of recreation for those who minister to the sick is not overlooked by the managers of hospitals beyond the seas.

We rejoice at these evidences of solidarity of hospital life, which neither distance nor climate can obscure. But they would never have been forthcoming if it had not been for the splendid pioneer work which has been done at home. If their own enterprise has prompted our kinsmen in distant parts of the Empire to take advantage of all that has been effected here to increase the comfort and promote the recovery of the sick and suffering, let us not forget that it is the hospitals at home which have provided the models, have set the high standard, and have largely supplied them with medical officers and with trained nurses. This, indeed, constitutes an additional claim on the part of the home hospitals to our sympathy and financial assistance. None of us know how soon we or our friends may have to seek the shelter of a colonial hospital, towards which we are not asked to subscribe a penny; and our duty to help our own institutions, which have been so materially the means of making these others what they are, is unmistakable. Possibly the idea obtains that during the present year less money has been diverted from the charities than in 1900; our columns to-day prove that the contrary has been the case, and that funds are more urgently required than ever. It is our pride and our glory that our hospitals in the Mother Country are maintained by voluntary offerings; and long may it be so. But unless every citizen, from the wealthiest to the poorest, helps according to his means, either directly by sending a subscription to a particular hospital, or indirectly by a contribution to King Edward's Hospital Fund for London, he will have neglected one of the primary obligations of the season, and can scarcely hope to enjoy the real happiness of Christmas Day.

A Corner of an Australian Hospital.

### CHRISTMAS IN A CEYLON HOSPITAL.

THE stranger entering "The Lady Havelock Hospital for Women and Children," in Colombo, Ceylon, on December 24th is confronted by greenery of all sorts and kinds in heaps, in festoons, and on the walls. Nurses—who are generally the essence of decorum and neatness—are now busily engaged making themselves dirty. Somebody breathes the magic word "Christmas," which explains all. "A happy midsummer to you," pants a voice from above. You gaze upward and encounter a smiling face shining equally with good humour and heat, not to mention dust. Deft hands are twining chains of a pretty trailing weed round the severe outlines of the barrack-room-like gas-pipes of each ward. The high decorator descends from her perch to get a fresh supply of "greens," returning with an armful in time to see her throne being cautiously dragged away by another busy worker who has watched her opportunity. A keen observer would see that, though fun and frolic are the order of the day, medicines are given and patients' wants are attended to—the stern hospital routine being little interfered with in reality. The whole aspect is a bright and graceful one, though the holly and the heavy evergreens of England are absent. The home decorators get scratched by the prickles and blackened by the smoke-begrimed leaves. In the colonies we work with less troublesome materials and are reddened

### SWEET CHARITY'S GUIDE TO CHRISTMAS GIVERS.

### GENERAL HOSPITALS.

**Great Northern Central Hospital,** Holloway Road, N.—Letters of recommendation are not required at this hospital; 159 beds are now open. Over 2,000 in-patients are treated annually. The hospital is unendowed, and the reliable income is quite inadequate to meet the necessary expenditure, over £10,000 being required annually from voluntary sources. The receipts of the hospital have been adversely affected by the competition of the war funds, a debt of £11,000 has consequently been incurred. Special precautions are taken here to restrict the benefits of the charity to the sick poor. Secretary, Mr. Lewis H. Glenton-Kerr.

**Guy's Hospital,** London Bridge, S.E.—At the present time Guy's Hospital is in urgent need of a capital sum of £180,000 towards a renovation and building fund from the benevolent in order to bring it to the highest state of efficiency and usefulness, and £25,000 per annum must also be obtained from voluntary sources to supply the deficiency in the income necessary for the maintenance of the complete establishment. The number of beds for in-patients is 652; the number of in-patients accommodated in 1900 was 7,320; the number of out-patients attended 124,262, and the number of maternity cases attended in the patients' own homes was 3,413. Treasurer, Mr. H. Cosmo O. Bonsor; Superintendent, Dr. E. C. Perry.

**Hampstead Hospital,** Parliament Hill, N.W.—During 1900 there were 312 in-patients, including 78 accidents and emergency cases. There were also 2,687 attendances in the out-patient department, including 324 minor accidents. The expenditure during 1901 will considerably exceed the income, and the Council earnestly appeal for additional help.

### IN A CEYLON HOSPITAL.

rather than blackened, roads in Ceylon being nearly all brick-red and the vegetation covered with red dust.

The patients, varying in hue, through all the shades of brown, from yellow to black are mildly interested; it would be against the principles of an Eastern people to be more than languid spectators. One misses much the lively interest and quaint suggestions of the dear home patients, though the Singhalese, Tamil, and Eurasian nurses are enthusiastic enough. On Christmas Day there is subdued expectation in the air, vague rumours of a present for each—both nurses and patients—are floating about. The great function of the day is to take place at 5 P.M., when the intense heat will be a little abated. Hot, dirty, and tired workers pass to and fro all day, arranging and rearranging everything. Work-a-day blue quilts are removed, and bright turkey-red ones substituted. The babies—known as "wee brownies"—are decked out in all the colours so dear to the Oriental heart and eye.

At 2 P.M. a huge wooden tub makes its appearance, borne by two coolies with great solemnity, and placed, upside down, between the large wards, that all may see and hear. Another tub follows, and is placed on the top of the first one. While the organisers of the plot are employed elsewhere, a scantily-clad coolie improves the shining hour by pouring the contents of two sacks into the top tub. They return to find that quite half of the bran has to be scooped out again to make room for the many mysterious packages which are now brought round in baskets to be buried. The unfortunate person responsible for the filling of the tub is well chaffed for giving extra work.

At length, a gigantic bran-pie comes into being. It is decorated with red, white and blue, and presents a very formidable appearance, which is enhanced when the whole structure is capped by a pie-crust. This crust is beautifully and wonderfully made out of brown paper stretched over a wooden frame. At 4 P.M. detachments of nurses begin to go off for a few minutes to dress. At 5 P.M. the visitors commence to arrive—"then the pie is opened and the brownies begin to crow." Every woman and child receives a present—cloths, jackets and dresses for all. Perhaps the most acceptable of all are the packages of the famous Ceylon tea beautifully done up and generously contributed by the planter friends of the hospital. Of course all the children get a toy each; every member of the staff is supplied with a curio. The night nurse whose thankless task it is to waken the weary day nurses is presented with a wooden scare-crow rattle. *Such* a noisy one!

The confined-to-bed patients are in the wards where they can see and hear all the fun. The others, in a large majority, are up and able to peep into the depths of the pie. The visitors seem to melt away, the holiday grandeur is quickly removed, and as the night rapidly sets in nearly all traces of the festivities are cleared away. The following day all the decorations are ruthlessly pulled down and rigid order is restored. The same bare appearance strikes one here as in England after the fun is over and the fragments of the festivities are cleared away; but for many days, perhaps years, the English Christmas customs participated in by patients of Eastern nationalities will be talked of and enlarged upon. On some occasions the time-honoured Christmas tree is the greatest function of the day; in fact, as far as circumstances will permit, Christmas is celebrated in Ceylon as it is in the dear Old Country.

### CHRISTMAS IN A BOMBAY HOSPITAL.

"*Madam Sahib! Tumárá Natal ká dêu, Tumárá burrá dêu, cubby ingá?*" (Your Christmas day, your feast day, when will it come?)

"Not for a month and a half, Armini," I answer, smiling at the wishful upraised face.

"*Kais-à luncba!*" (how long), replies the little Mohammedan.

"Shall I get a *buksheash?*" (present).

"Certainly. What would you like?"

"*Coops and sasers,*" is the prompt reply.

This will be the child's first Christmas in hospital. Rumma, her Hindu friend who was with us last year, has told wonderful tales of that happy time. Of the feasting, and of the marvellous tree that grew up suddenly in the children's ward, bearing on its branches, toys, oranges, and coloured *butties* (lamps) for fruit.

Armini frankly told Rumma that her statements were incredible. Nurse, however, confirmed them, and the Mem Sahib's word being above suspicion, the little girl could only wait and long for the happy day. It is here at last. "Why are they all so glad?" asks one native of another as the day nurses come on duty and pass with bright faces to their several

### SWEET CHARITY'S GUIDE TO CHRISTMAS GIVERS.

### GENERAL HOSPITALS—*Continued.*

especially new annual subscriptions, to meet this deficiency. The present hospital being totally inadequate to meet the wants of the neighbourhood, a building fund has been started, and plans are being prepared for the erection of a new hospital on a site which has been obtained on the top of Haverstock Hill. The benevolent are asked to assist in this work on behalf of suffering humanity.—Hon. Secretary, Mr. R. A. Owthwaite.

**Italian Hospital,** Queen Square, Bloomsbury, W.C.—The hospital is a general one, free to the sick and injured poor of all nationalities and creeds, and entirely dependent on voluntary support. Nearly 40 per cent. of the patients are British subjects. The year 1900 closed with a deficit of nearly £400 in income, and the numerous demands upon the charitable during the past twelve months have further affected the Hospital's revenue. Consequently, unless additional help is speedily forthcoming, some of the beds at present remain unoccupied for lack of funds, but with a comparatively small addition of income the hospital's full benefits would be available. Hon. Secretary, Cav. Luciano de Rin.

**King's College Hospital,** Portugal Street, Lincoln's Inn, W.C.—2,746 in- and about 19,913 out-patients were treated in 1900, and the expenditure exceeded the income by £2,000. Mr. Charles Awdry, Treasurer.

**London Homœopathic Hospital,** Great Ormond Street, W.C.—The urgent need of this hospital is an increase of annual subscriptions. The sum needed yearly for maintenance is £10,000, while the annual income is under £8,000. Its operations comprise not only general medical and surgical work, but various special departments, also an electrical department for Roentgen rays and the treatment of

### IN A BOMBAY HOSPITAL.

wards. "Oh, it's the day their God was born," is the reply. "Why are they all so glad?" That must be the thought in many a mind as the nurses tend their sick, dressing each woman and child in a new jacket or frock, adorning the necks of the little girls with strings of bright blue beads, and their hair with ribbons of the same colour

Presently the sister in charge comes round with a bright smile and a kindly word for both nurses and patients, and a trifling gift for each of the children. How pretty the wards look! The floors are beautifully tiled in bright mosaic, coloured pictures adorn the walls. In convenient places are hung wire baskets filled with growing ferns and moss. On the tables, Japanese trays hold the medicine glasses, quaint inkstands of Indian workmanship are on the writing boards, the brass rulers and paper-weights reflect all near objects, and every available vase is filled with flowers, scarlet and cream hibiscus, roses, eucharis lilies, pink and white oleander in lavish profusion, while the golden sunshine streams in at the open windows, making it difficult for those who hail from dear old England to believe that it is really Christmas Day. The cots, as bedsteads in India are always called, are painted red and covered with red blankets.

The patients represent many nationalities—Hindus of various castes, from the priestly Brahmin to the low-born sweeper. Bene Israel (native Jews) and white Jews from Bagdad, Mussulmans, Parsees and Arabs, Japanese, Chinese and Africans; all the nations of the earth for whom the angels brought "good tidings" on that first Christmas night are to be found within our hospital walls.

The doctors come late this morning and go through the wards expeditiously, avoiding unnecessary work. One operation, however, cannot be delayed; but it is over in time for the surgical nurses to join the rest in their pretty bungalow at eleven o'clock for breakfast, the Hindu nurses taking charge of the wards meanwhile.

To-day, sisters, charge-nurses, and probationers breakfast together. The long table laden with good things, decorated with a wondrous wealth of tropical flowers and ferns, and surrounded by happy faces, is a pleasant sight. Dusky servants clad in snowy raiment, with red and gold turbans and cummerbunds, wait on the party with quiet gravity. "*Kissmis*" is a *burra dêu* to them too, for is it not a day on which the Sahibs give *buksheash*!

Later on the patients come in for their share of good things, their Excellencies, Lord and Lady Northcote, having sent a large sum of money to be spent for their benefit, but the great event of the Christmas week is the children's treat at the Petit Hospital. The surgical ward is cleared for the afternoon, cots and lockers finding a temporary place in the roomy verandahs. All the morning sisters and nurses have been hard at work, but at four o'clock the preparations are declared complete, and the visitors arrive.

The walls of the ward are adorned with gay bunting, whilst strings of small coloured flags cross the room at every angle, and in the centre stands the beautiful Christmas Tree. Flowers are in abundance, of course, but they scarcely seem to be needed, for the children themselves suggest a parterre as they sit on either side of the strip of grass matting on which is spread their Christmas feast. Sister's store cupboards have been heavily indented on and every child is clad in a new garment, whilst those whose crowns are not clean shaven have had their hair oiled and brushed till it shines again. Silver and glass bangles sparkle on the children's tiny wrists and ankles, and quaint little ornaments adorn their ears. Here and there one notices a small girl wearing a nose-ring, and we know by that, and the strings of black beads round her neck, that she is a Hindu child-wife.

The older children have been brought across on stretchers from the Jamsidji Hospital, Armini amongst them. Little Orientals take their pleasures somewhat soberly, but indicate by the dilatation of their large dark eyes and the smile on the lips of each, how much they are enjoying themselves.

Look at that wistful-looking group near the door. They are the children of a Brahmin nurse. You may tell their *ját* (caste) by the way in which the girls wear the *lugádá*, exposing the calf of the leg to view. Their hair is tightly drawn back from the brow, stiffly plaited and arranged at the back of the head in a shape of a tea-pot handle. A flat gold ornament keeps it in place at the top.

It is of no use asking these high-caste children to sit down with the rest. They resolutely refuse dainty after dainty with an emphatic "*Nako!*" The sweets, however, prove too strong a temptation, and, having eaten them, they become so thirsty that they forget their prejudices and ask for tea!

Now it is time to light the tree. Who shall describe the babel that ensues, when in several languages cries of wonder and admiration arise? Dear little ones! Will you ever know the real meaning of the Christmas Tree and the *Sahib lôke's*

### SWEET CHARITY'S GUIDE TO CHRISTMAS GIVERS.

### GENERAL HOSPITALS—*Continued.*

lupus. Last year 1,128 in-patients were relieved, and the out-patients' consultations numbered 36,795. An earnest appeal is made for new annual income. Secretary-superintendent, Mr. G. A. Cross.

**London Hospital**, Whitechapel, E.—This is the largest voluntary hospital in this country, and stands sorely in need of help. Important and much-needed alterations are being rapidly completed to improve the accommodation for those who are obliged to resort to it in times of sickness and accident. Under the able leadership of the Hon. Sydney Holland, as chairman, the committee are striving to make this hospital fully equal to the demands made upon it. The work is admirably carried out, but its range is much restricted by the pinch of poverty. We strongly urge our readers to help this great charity of the East End. Full information about the institution may be had from the House Governor and Secretary, Mr. G. Q. Roberts, M.A.

**Metropolitan Hospital**, Kingsland Road, N.E.—The need for this hospital in the poor and densely populated districts in the midst of which it is situated is shown by the fact that both the in- and out-patients have been steadily on the increase for several years past, the numbers treated last year being: in-patients 964 and out-patients 33,708, the attendances of the latter numbering 106,896. There is a debt at the present time of over £3,500, incurred during the past four years. The hospital has accommodation for 160 in-patients, but hitherto the committee have only been able to maintain 76 beds; the demand on these was so great that the committee recently decided to open two more wards, making in all 100 beds now available, feeling assured that

*burra dēu* ? Who will tell you of the Holy Christ child and the first Christmas day ?

By the time the tree is stripped every child has its hands full of presents, and the festivities have been considerably enhanced by the addition of a band! A big drum, concertina, and musical box with sundry trumpets and whistles discourse *sweet discords.* Armini is radiant. The best doll has fallen to her share. She has got the "coops and sasers," with spoons to boot, and a toy piano besides. No wonder that when once again in her own little bed at the Jamsidji she should say as she puts her soft arm round nurse's neck, *Mem Sahib tumárá burra dēu bante achā* "! (Your feast day is very good.)

### CHRISTMAS IN A PUNJAB STATION HOSPITAL.

CHRISTMAS out in India always seems a season in which to feel qualms of home-sickness, even if one does not suffer from the complaint at any other time; and the majority of people who can get leave always try and go away into another station for change of scene, or into camp for a few days' shooting. We always try to brighten the unfortunates who have to stay and make the best of things in hospital, more particularly "Tommy Atkins." British soldiers are nothing if not sentimental (at least on foreign

A Ward in a Fiji Hospital.

### SWEET CHARITY'S GUIDE TO CHRISTMAS GIVERS.

### GENERAL HOSPITALS—*Continued.*

by so doing they would receive increased support from the charitable public. Secretary, Mr. Charles H. Byers.

**Middlesex Hospital**, Mortimer Street, W.—The in-patients for last year numbered 3,758, and the out-patients 48,911. The income from all sources, including legacies, was £31,279, and the expenditure £34,322; deficiency on the ordinary income and expenditure, £5,092 The cancer wards are a distinguishing feature of the hospital, and their extra nursing, costly treatment, and unlimited dietary add largely to the expenses of the hospital. Towards the extension of this department, by removing it from the main body of the hospital, and building a new wing, £9,000 is still needed. Secretary-Superintendent, Mr. F. Clare Melhado.

**North-West London Hospital**, Kentish Town Road, N.W., was founded in 1878, and is the only institution of the kind in the north-west district. It has 53 beds, and last year there were 494 in-patients and 15,019 out-patients. Secretary, Mr. Alfred Craske.

**North London or University College Hospital**, Gower Street, W.C.—This hospital was founded in 1833, and during the past 68 years has treated over one million and a-half patients. In 1900 2,696 in-patients and 37,453 out-patients and casualties were treated The attendances made by the out-patients and casualties amounted to the enormous total of 131,086. Nearly 800 surgical and other appliances were supplied, and 233 patients were sent to convalescent homes. It is estimated that when the whole building is completed, the total annual expenditure will be between £28,000 and £30,000, and, as the reliable income from all sources is only £8,000 per annum, it will be seen that the adequate support of the enlarged and modern

### IN A PUNJAB STATION HOSPITAL.

service), and many are the photographs and small souvenirs from their best girls at home, which are produced from under pillows after the mails have come in.

On Christmas Day itself little can be done, as a rule, except give cards and good wishes all round, because the few people left in cantonments are busy all day with regimental and social engagements, but generally some of the officers and the chaplain hand out cigars and cigarettes for the men allowed to smoke. The day after, however, in our station, we generally give the orderlies and convalescents an entertainment. Last year it took the form of a tea and smoking concert for all allowed up in our wards, and for as many of the men from the other block as we could find room for. I do not think we had a spare moment from early morning. As we had some bad cases of enteric, of course no noise could be allowed in the sisters' block, but, with the permission of the doctors, we used a ward on the other side.

One sister was on night duty, and another busy in the wards, so one only could be spared to get everything ready. However, she had a small army of willing helpers in the orderlies off duty, and in a few hours the bare ward had been turned into a very festive scene, with lots of greenery which the men wreathed very prettily around all sorts of seasonable mottoes, pictures, and a big Father Christmas, all of which had seen their first Christmas in a London hospital, and have since done duty in various Punjab stations. At one end a charming drawing-room was made with rugs, curtains, easy chairs, from the sisters' quarters, and any number of plants; and when a piano arrived from one of the messes, that part of the room was most fascinating.

At the other end long tables were simply groaning under huge sugar-covered cakes, jam, piles of bread and butter, and various additional good things. On each cup (to economise space, regimental tables being narrow) was a large Christmas card with the names of the performers at the concert written on the back, and on these cards cigars and cigarettes were piled up; they had rather a pretty effect when viewed from the distance. The cutting up of the bread and butter had kept one lady helper and one orderly corporal hard at work for hours, but they must have felt rewarded when they saw the rapidity with which it disappeared. We always have the tea made in teapots, and the men put in their own milk and sugar, which they much appreciate—tea in barracks being boiled by the gallon or so is anything but delightful and home-like.

We had invited the men for 4 P.M., and by that time groups of convalescents, as brushed-up and smart as the exceedingly ugly hospital kit will allow them to look, were waiting at the doors. We never allow any of the orderlies to attend on the patients at tea-time, but they have their own table and are guests; the hard work of running over to the cook-house and replenishing the teapots being done by two or three able-bodied British subalterns, whom we always press into the service on these occasions.

At 5 P.M., when the men had finished tea, our station visitors began to arrive—the officers and ladies who had promised to sing or play, and a great many more who came to listen; we had a tea-room for them in a corner of our verandah—prettily decoated with flowers. I did not allow them much time for chatting out there, as by 5.30 our men were all in their places and the concert began.

We were lucky enough to have three violins, and as there happened to be a good deal of talent in the station just then there was no lack of singers, and one sapper's wife, looking charming in a cream frock, quite brought down the house in "May Morning." I think the greatest applause was given by the men to an Irish doctor, who always goes very strong at these shows, and fairly convulsed us all with his comic songs; what he lacks in voice he makes up for in expression. There had been so few delays that by 7.30 we had gone through 21 items, in addition to encores which the men had insisted upon, and I am sure that our good-natured performers deserved the cheers with which the Tommies bade them good-bye.

After all our friends had driven away packs of cards were given out; and the men finished up a pleasant evening by a sing-song all to themselves. One of our orderlies, having been a music-hall singer when in civil life at home, was in much request, and I heard vociferous applause in the distance.

I went over to the first ward in one block in which were the bad cases, and there found a very different scene—the sister on duty without a moment to spare, amongst bad enteric and pneumonia patients—the delirium of two taking curious forms, one saying his prayers at intervals, and his companion in the next bed, also wildly delirious, using a very choice collection of "cuss words!" However, they both recovered and were sent home, so we will hope that they are having a happier Christmas this time.

### SWEET CHARITY'S GUIDE TO CHRISTMAS GIVERS.

### GENERAL HOSPITALS—*Continued.*

hospital will necessitate a much larger amount of voluntary assistance. Secretary, Mr. Newton H. Nixon.

**Poplar Hospital for Accidents**, Blackwall, E.—Situated amongst a teeming population of poor hard-working people in a district which may be called the "workshop" as well as the "port" of London, this hospital is doing an excellent work. The demands on this institution of late years have greatly increased, and consequently further subscriptions and donations are very necessary. Chairman, the Hon. Sydney Holland. Secretary, Lieut.-Colonel Feneran.

**Royal Free Hospital**, Gray's Inn Road, W.C.—Having no endowment, this hospital is entirely dependent for support on the subscriptions of its governors and the voluntary donations and bequests of its friends. It admits into its wards over 2,000 poor sick persons annually, besides administering to more than 30,000 out-patients who resort to it, not only from the immediate neighbourhood, but from all parts of London and the suburban districts. As is indicated by its title, admission to the hospital is entirely free; no subscribers' letters are required, and no charge whatever is made to the patients. The relief thus afforded is effected at a cost of about £12,000 per annum, while the reliable income does not exceed one-third of that sum. Secretary, Mr. Conrad W. Thies.

**St. George's Hospital**, Hyde Park Corner, S.W.—This institution contains 351 beds, and during 1899 treated 4,106 in-patients, 13,087 out-patients, 12,556 casualties, and 499 maternity cases. It has been recently remodelled throughout and is one of the most deserving and best administered of hospitals. Secretary and Superintendent, Mr. C. L. Todd.

### CHRISTMAS IN A HONG KONG HOSPITAL.

CHRISTMAS in the tropics! A fairly easy subject to write about, one would think; in fact, I almost hear my readers say, "Yes, a Christmas abroad has so many points; it must be so very different!" "There's the rub; it *is* so different. Imagine, if you can, a Christmas, sans snow, sans fires, sans holly, or any of the usual adjuncts of that festive time at home, spent in a place where the people at the best consider the gala day a species of "foreign devil joss," which is not altogether unworthy of their attention since it may result in a "cumshaw" (present), pecuniary or otherwise.

Let us take a typical Christmas day in Hong Kong, and in the blazing sun, with the temperature in the shade at 80° Fahrenheit, proceed in a hooded jinricksha or a covered Sedan chair to the Government Civil Hospital to inspect the preparations made for the entertainment of its very cosmopolitan inmates.

The top flat, consisting of two large, two small wards and the operating theatre, is the centre of attraction, for here the concert, the tree (by no means perfectly straight, but sufficiently loaded to prove attractive) and the refreshments pander alike to the tastes of the artistic and the thirsty. Most

A Convalescent Hospital in Camden.

### SWEET CHARITY'S GUIDE TO CHRISTMAS GIVERS.

### GENERAL HOSPITALS—*Concluded.*

**St. Mary's Hospital**, Paddington, W.—Very heavy work is thrown upon this institution owing to the large area of scattered poor whom it has to assist. It is not so well supported as it should be, in spite of, or rather because of, its many wealthy neighbours. The board of management, therefore, urgently appeal for further support. The special wants of the hospital are: (1) New annual subscriptions; (2) donations for the endowment of beds and cots. The hospital is free, and no urgent case is refused admission. Secretary, Mr. Thomas Ryan.

**Westminster Hospital**, Broad Sanctuary, S.W.—Out of an expenditure of £20,000, less than £3,000 is assured to this charity, so that about £17,000 has to be made up each year in subscriptions, donations, and legacies. The work of the hospital in 1900 included the treatment of 2,363 in-patients 22,254 out-patients and casualties, and 226 lying-in women. The hospital does an immense service to the country by training a large number of excellent nurses. Secretary, Mr. Sidney M. Quennell.

### PROVINCIAL HOSPITALS.

**The Birmingham General Hospital.** (Founded 1766).—The new building of this hospital was opened by H.R.H. Princess Christian, on behalf of H.M. Queen Victoria, on July 7th, 1897, and contains 346 beds. To meet the increased accommodation provided a larger income is necessary, and an earnest appeal is made for new and increased annual subscriptions or donations. House governor, Mr. Howard J. Collins.

### IN A HONG KONG HOSPITAL.

tropical hospitals are built with a broad, covered, stone verandah, serving the dual purpose of protection from the sun's glare and the torrential showers of the monsoon.

I remember my first Christmas Day decorating the festooned archways of these verandahs with Chinese lanterns—made in Germany!—some two or three hours before twilight and rejoicing in the fact of the candles being all in readiness. Mistaken zeal, for lo! when they came to be lighted, the heat of the sun had reduced them in appearance to miniature copies of the Tower of Pisa.

But the mystic hour of 3.30 approaches, and the native male nurses, with highly polished faces, neatly dressed pig-tails, spotless clothing, and a new blue ribbon—the Government badge—tied round their stockings, emerge after their labours in wreath-making to take part in the fun.

These "Boys" as they are called (being perennially young they answer to that name until even the age of 40 or 50 is reached) take an active interest in all the proceedings, and vie with each other in bringing the first news of a far off gleam of the scarlet uniforms with a white crown, which indicates the arrival of His Excellency the Governor or some of his suite. Another will rush in with the news that "The No. 1 soldier-man" (the General) or "the top-side joss-pidgin man" (the Bishop) has been sighted, and then the influx of visitors and the distribution of programmes and fans become general.

Follow with the throng into the spacious seamen's ward, cleared for the occasion, where the patients have already been placed. It is very gaily decorated with bunting, and the platform for the performers is a mass of palms and exotic blooms.

In the rear, raised tiers are occupied by the wives and children of "The Boys" and by the juvenile members of the diocesan schools, invited for this occasion only, who come determined to enjoy every item of the programme, the evolution of which has proved such a Herculean task, for the nationalities range from the heathen Chinee to that of the Norwegian or Russian Finns, and the difficulties attendant upon catering for these make one wish that the introduction of a little mortar from the Tower of Babel into one's composition had been possible.

"Music would be the very thing." I hear someone say. Yes, one could imagine all the world meeting upon that point, but Heaven defend us if China or India took the lead, for their musical instruments produce a combination of the proverbial German band and the bagpipe. So, chiefly for the European inmates, songs and carols are sung and a little instrumental music indulged in, until our childhood's friend, the mock-conjuror, comes in and elicits mirth from all. See him balance a ball running round upon the opened umbrella he is twirling so rapidly, and watch the expression upon the faces of the marvelling audience. Then let him hand the umbrella to his confederate who in closing it reveals the ball depending from its string, and the yells of laughter which greet his exit from the stage will tell you that the performance has appealed to every unit of the crowd. The strains of the National Anthem cause a general uprising, and the sisters in their pretty "butcher-blue" uniform flit theatre-wards to see that no item of the tea provided for the guests is lacking.

Little do the latter dream that their gaily-draped refreshment room is ofttimes the scene of heroic resolve or tragic despair upon the part of those very patients whose Christmastide they have endeavoured to brighten by their presence and gifts. But to the distribution of these it is time to turn our attention, for the tree under a sister's direction is being rapidly denuded by willing and capable hands. Every patient receives at least one present, and as far as possible individual need and liking are considered, so there is a brilliant yellow puggaree for the phthisical Indian whose bright eyes grow brighter at the prospect of possession. Towels, and gay pocket-handkerchiefs, especially if flanked by a cake of soap, find favour in the eyes of his more convalescent brethren, while woollen scarves and Dogger-bank caps are eagerly sought by the police of all ranks as a protection against inclement weather.

Pipes, dolls, toys, sweets, shoes, petticoats, flannelette coats for the women, frames, tobacco, and purses go to make up the heterogeneous whole, and the hearts of the Amahs—female nurses—are gladdened by the welcome gift of the ever-necessary umbrella or a pair of goloshes.

The forlorn looking tree, shorn even of the tinsel trifles which enhanced its effectiveness, is now abandoned, and the patients depart to their respective wards to discuss alike their "cumshaws" and the sufficiently attractive meal which awaits all those convalescent enough to partake of it. Bonbons abound, and there is something at least of the true Christmas spirit in the heart of the burly policeman, that dreaded myrmidon of the law, who has selected the bulkiest cracker and is pressing it with gentle insistence upon the shy little maiden

### SWEET CHARITY'S GUIDE TO CHRISTMAS GIVERS.

### CONSUMPTION HOSPITALS.

**Bournemouth National Sanatorium for Consumption and Diseases of the Chest.**—Patients in humble circumstances who are convalescent, who require further medical treatment and change of air, or who are suffering from an incipient form of the disease, are cases for the relief of which this institution was founded. The "open-air" treatment is carried out completely in all suitable cases. There is at present only accommodation for 31 men and 31 women, but to meet demands, building extensions are being provided to add 10 beds, and for this purpose about £1,000 is still required. The committee earnestly solicit liberal support, as this national sanatorium is almost entirely dependent on voluntary contributions. Secretary, Mr. G. Lowe Riddett.

**Brompton Hospital for Consumption and Diseases of the Chest,** S.W.—This hospital is well known as one where patients are well cared for, and every effort is made to brighten their clouded lives. The committee appeal for new subscribers to replace those removed by death and other causes, and for assistance to enable them to erect a much-needed "country branch and convalescent home," the site for which has been already secured at Heatherside, near Bagshot, and where the "open-air" treatment will be largely practised. About £40,000 is required for this purpose. Gifts are also asked for towards the annual Christmas tree. Secretary, Mr. William H. Theobald.

**North London Hospital for Consumption and Diseases of the Chest,** Mount Vernon, Hampstead, N.W., and Fitzroy Square, W.—Particular attention is drawn to the urgent need of completing the hospital at Mount Vernon whose one-legged condition appeals pathetically to all. Perhaps he is thinking of his family at home and of the time when, foreign service ended, he may look forward to a Christmas spent with them|; perhaps it is this thought which engenders a suspicious kind of sneeze, demanding a vigorous application of his new pocket handkerchief in the region of his eyes and nose, but onlookersare told that "sitting in a draught —I would there had been one—has given him a confounded cold." So we pass on, thinking none the less of him for such a shallow pretence.

Games, smoking, and feasting wind up the evening, and at 9 P.M., when the lights go out, exit Father Christmas until another twelve months have passed.

### CHRISTMAS IN A CAPETOWN HOSPITAL.

IN writing of Christmas time in this country one cannot help thinking of many Christmas Days in dear old England. I suppose there were damp and foggy days at that season of the year, but upon looking back I seem only to remember opening my eyes when daylight dawned upon "a white world." How beautiful even the chimney-tops of dear grimy old London appeared when covered with a mantle of snow! How gaily we went from ward to ward singing carols, and how happy were the little faces in the children's ward, the picturesque little figures in their scarlet jackets and the wistful eyes fixed upon a bulky stocking at the foot of each cot; how cheerily we exchanged "Merry Christmases" with all and sundry, and gave a word of sympathy alike to those who were inclined to be tearful from home-

The Children's Ward in Bendigo Hospital.

### SWEET CHARITY'S GUIDE TO CHRISTMAS GIVERS.

### CONSUMPTION HOSPITALS—*Continued.*

Hampstead, thereby increasing the accommodation from 100 to 140 beds. For many months past the duties of the medical staff have been of an exceptionally difficult and trying character. To be compelled to send away—not occasionally, but daily—applicants who are in every respect eligible for indoor treatment, and whose lives might be prolonged, is a terrible experience. But the difficulties do not end here. For those who are approved—and there are now nearly 250 such waiting their turn to be admitted—there remains the long and weary wait of at least twelve or fifteen weeks, during which time the disease which has attacked them pursues its course with such deadly effect that many who were not beyond hope of cure when approved, are hopelessly ill when they are received. It is to remedy these unsatisfactory conditions that help is required. Of the £12,000 needed to complete the hospital, £3,000 has yet to be subscribed.—Secretary, Mr. W. J. Morton.

**Royal Ho-pital for Diseases of the Chest,** City Road, E.C.—The expenditure at this hospital exceeds £8,000, towards which there is an annual subscription list of some £2,400, and dividends amounting to £135. £4,000 is urgently needed to build and furnish a nurses' home. Donations will be gratefully acknowledged by the Secretary, Mr. John Harrold.

**Royal National Hospital for Consumption and Diseases of the Chest,** Ventnor, Isle of Wight.—The hospital now accommodates 153 patients. Every bed is occupied, and 227 candidates are waiting their turns for admission. As the expenses exceed the assured income by £4,000, the com-

### IN A CAPETOWN HOSPITAL.

sickness, and to the majority whose faces beamed with delight at the prospect of spending a real Christmas in 'orspital.

But in sunny South Africa we do not look for such an outward and visible sign of Yuletide as the appearance of Jack Frost; we expect what we generally receive, *i.e.*, a typical midsummer day. Nor can we decorate in the lavish way affected at home with the seasonable holly, we have so little of it, and what we have must be put aside for another use, for has it not been spoken of by the coloured maid as "the pudding flower?"

To really enjoy a visit to a South African hospital at Christmas time, I must induce you to follow me, at least in spirit, to Table Bay, and imagine, if you can, this lovely bay filled with ships of every kind—transports, which are waiting to convey poor, sick Tommy back to some of "the little things he's left behind him"; mail boats, also homeward bound; and still smaller, though more picturesque, craft of all sorts. There lies Table Mountain, its colour changing, as it were, with every mood, from blue to purple, and from purple to warmest flame-colour; and above, a sky of deep azure with scarcely a cloud to be seen, unless that lovely little piece of cotton-wool which is floating towards the mountain can be called by that name.

Then, as I wend my way through the streets of Capetown, I cannot help remarking upon the striking and cosmopolitan crowd, such as you would find nowhere else but in the Egyptian capital. All are apparently on pleasure bent; the electric trams are filled with "all sorts and conditions of men" and women, most of whom are going to spend the day at the houses of friends in true South African fashion, which includes plenty of tennis, and plenty of cooling drinks; indeed, one soon becomes accustomed even to the idea of plum pudding and mince pies, followed by unlimited strawberries and cream! And here we are at last at the gates of an imposing looking building, overlooking the bay; and I hasten to introduce to you the New Somerset Hospital, which for many years has been the general hospital of Capetown. It is not easy to realise that we are 6,000 miles from home, for once inside this beautiful hospital, the nurse must feel thoroughly at ease; and as it is now afternoon and we are told that the Christmas tea and tree are going on in the children's ward, we turn our steps in that direction.

The "Lady Loch" ward is at all times a charming sight, but never so attractive as to-day. Loving hands have been at work decorating everything with flowers; and there we see the time-honoured Christmas Tree, ever dear to the childish heart, and indeed to the hearts of many of us who ought, perhaps, to have "put away childish things." As the ward is filled with sunshine, the little coloured candles have not been lighted, but this does not trouble the small persons chiefly concerned; their happiness is too great to be damped by trifles. Presently, from some mysterious source, presents of all kinds and suited to every need are produced, and great is the joy of the children and of the adult convalescents who are well enough to partake of the good things provided. But at this point we leave the infants to calm down before being settled for the night; and take a peep at the other wards.

One glance into the men's ward assures us that they have not been forgotten. Each patient has been presented with a new pipe and some tobacco, and what could be more soothing to the mind of the average male than a timely gift of "My Lady Nicotine"? All around, one sees evidences of the tender care with which the wards have been decorated; and in the women's wards the patients are enjoying the sweet-meats provided for them, and wearing the dainty buttonholes sent to them by some kindly lady. As we move along with the other visitors, one's thoughts turn involuntarily to Him, Whose Birth we are commemorating and Whose Life was spent in doing good to the sick and sorrowing. We think, too, of the sad sights which Christmas in the old country has often revealed to us, the intense suffering of the poor, and the drunkenness and sordid misery of their lives, and we thank God that in South Africa we have no such poverty, and that His glorious sunshine makes life a brighter and more joyous thing than in England.

But it must not be supposed that the nursing staff has been overlooked. Quite a grand evening is provided for them, to which their friends are invited, and a concert is given, followed by a supper. The friends of the hospital whose contributions are devoted to this purpose, must feel happy to know that those whose lives are given to the care of the sick have some good times provided for them. And once again one's thoughts go back to the old days. Can one ever forget one's first Christmas in hospital? The excitement of it all, mingled with the home sickness, and, oh! such aching feet! As we wish our friends "Good night" at the gates of the hospital, we forget that yonder is the Southern Cross, and only re-

### SWEET CHARITY'S GUIDE TO CHRISTMAS GIVERS.

### CONSUMPTION HOSPITALS—*Concluded.*

mittee appeal for additional annual subscriptions and donations. Secretary, Mr. Ernest Morgan. London office, 34 Craven Street, Charing Cross, S.W.

### LYING-IN HOSPITALS.

**City of London Lying-in Hospital**, City Road, E.C.—During the past year more patients have been treated than in any previous year, and funds are needed to carry on the general work of the hospital, and also to defray the cost of building new dormitories, which the committee have now provided for the nurses and pupils at an outlay of over £4,000. Secretary Mr. R. A. Owthwaite.

**Queen Charlotte's Lying-in Hospital**, Marylebone Road, N.W.—This hospital received 1,183 patients into its wards last year, and, in addition, attended 971 patients at their own homes. The enlargement and renovation of the hospital have been completed, and much-needed additional accommodation is now available. Her Majesty Queen Alexandra has accepted the office of Patron, and has become an annual subscriber. A feature of the work during the past two years has been the admission as patients of the wives of soldiers and sailors sent on active service to South Africa. The expenditure amounts to £5,000 yearly, while the only reliable income amounts to £2,000. Contributions towards the deficit of £3,000 and towards the £7,000 still needed for the building fund are, therefore, earnestly solicited. Secretary, Mr. Arthur Watts.

### HOSPITALS FOR EPILEPSY AND PARALYSIS.

**National Hospital for the Paralysed and Epileptic** (Albany Memorial), Queen's Square, W.C.—The total number member that our hearts are with our dear ones over the seas, and with those whose homes have been bereft during the past year, and whose sad thoughts must constantly turn to lonely graves on the veldt where son or husband sleeps his last sleep. We breathe a silent prayer that to this land, as to the hearts of men all over the world, the message of old may be sent of "Peace on earth to men of good-will."

### Christmas in a Pretoria Hospital.

It is midsummer, and the scent of roses is on the air. Yet this is Christmas night in South Africa. A hot, steamy night, bringing little refreshment after a hot, steamy day. The hospital is a long one-storeyed building, made for a boys' public day school, and consisting of class-rooms, large and small, and a gymnasium. The Boers used it as a prison for British officers, and in one of the wards is still to be seen the trap door in the floor through which several enterprising men made their escape. The class-rooms make excellent wards, some with 8 and some with 16 beds, and altogether 130 patients can be accommodated. There is a first-rate theatre, well equipped, and with many useful adjuncts, such as an abundant supply of Roentgen Ray apparatus, an elaborate electric battery, and a dark chamber for examining eyes improvised out of a black tarpaulin wagon cover. The whole hospital is well lighted by electric light. Many men come in here for operations which are necessary to restore them to efficiency in the field. Radical cures are frequent, so is operative treatment of hammer toes and other distortions of the foot due to heavy marching. In the case of mounted infantry hæmorrhoids have often to be removed. This, therefore, is the surgical section of the Schools Hospitals, and it has an excellent record of successful cures.

In the building itself there are only wards, an office, a store room, and the theatre. All cook- and wash-houses, sanitary arrangements, and so forth, are in temporary buildings in a compound behind. There are just two wards set apart for enteric and dysentery cases who have overflowed the fever and medical sections in the girls' school buildings one or two streets away. Otherwise, the inmates are all healthy surgical cases, men of picked physique between the ages of 18 and 40, with corresponding cheerfulness and energy. Some days ago they determined to spend a merry Christmas, and since then the talk has been of nothing but of food and decorations. It has been rumoured that chicken and fresh vegetables will be provided all round for the patients' dinner, and geese for the staff. The orderlies are to have a pint of beer apiece at the expense of their grateful country—a very enjoyable change from enforced teetotalism. Tinned plum puddings in large numbers will be provided from the Field Force canteen.

As for decorations, a few days' hard work have transformed the hospital into a garden. The men are experienced in decorating barracks and most ingeniously they set to work to evolve an elaborate display out of the simplest materials. Coloured tissue paper is the chief medium of ornament. The doctors have kindly provided the money and the sisters have ransacked every shop in Pretoria until there is not a bit of coloured paper left for sale. Festoons and streamers of pink, green and white are strung across the walls and ceilings. In one ward, on the night sister unwarily turning up the central lamp a shower of paper roses descended on her head. The orderlies have been roaming over the commons round Pretoria and have brought in armsful of a trailing, feathery green like asparagus—only it has thorns. This has been twined round the pillars of the gymnasium ward, and disguises jumping bars and other mechanism. Another ward has a dado of green frosted with some white powder to recall far-distant snow. Placards, handsomely lettered and coloured, hang on the walls proclaiming suitable sentiments, and calling down blessings on all the staff. "Long Life to Colonel N.," "Success to Major C.," "God Bless Mr. G., Tommy Atkins' beau ideal of a Gentleman." This refers to the clever civil surgeon, whose operative skill has given so much relief. There are several compliments to the sisters and orderlies, and the R.A.M.C. badge—a serpent climbing a pole surrounded by a laurel wreath —is handsomely represented in many colours. Several legends of course evoke blessings on "The Queen." One of these, indeed, remained on the wall long after death had claimed the Queen it was put up for. There were differences of opinion about taking it down some three months later, as many thought it might hang there permanently in reference to Queen Alexandra. In front of a stimulant cupboard hang two highly-illuminated texts—the one proclaiming "More Power to Lord Kitchener," the other "Cead

### SWEET CHARITY'S GUIDE TO CHRISTMAS GIVERS.

### HOSPITALS FOR EPILEPSY AND PARALYSIS

*Concluded.*

of beds provided at this hospital and its Finchley branch is 200, but the accommodation is still painfully insufficient to meet the requirements, as the large majority of patients are unsuited to general hospitals. Many urgent cases are always waiting for beds. Besides the hospital for treatment, there is a pension fund for the incurables. The annual expenditure is £18,500, of which upwards of £10,000 must be raised in benefactions. Director, Mr. B. Burford Rawlings.

### CHILDREN'S HOSPITALS.

**Alexandra Hospital for Children with Hip Disease,** Queen Square, Bloomsbury, W.C.—The necessity for this hospital for hip disease may best be shown by the fact that nearly three-fourths of its patients come from other London hospitals, the reason being that no general hospital can retain a patient for two or three years, as is not infrequently done in this hospital. £1,000 is urgently needed for maintenance. Secretary, Mr. Stanley Smith.

**East London Hospital for Children and Dispensary for Women,** Shadwell, E.—It will be readily understood that the struggle for existence in such a neighbourhood as Shadwell is very great, and for this reason the committee of this Hospital for Children appeal to the residents in wealthier districts for funds to enable them to help those who are unable to help themselves. The seaside branch at Bognor (28 beds) for convalescent patients materially increases the usefulness of the charity, and at the same time its need of support. Secretary, Mr. Thomas Hayes.

**North-Eastern Hospital for Children,** Hackney Road, Bethnal Green. — Founded 1867. The main part (the

### IN A PRETORIA HOSPITAL.

Mille Failthe." This latter sentence had to be carefully spelt by an Irishman to the Scotchman who carried it out. The night sister, searching in the dark for brandy, got into difficulties with these texts, until a six-foot warrior sprang from his bed, regardless of veldt sores on his legs, and held aside the encumbrances.

The fun of decorating is over. Christmas night has come, and most of the men are sleeping as only soldiers can sleep when lately "off the trek." A solitary sheet is all their covering, and even that seems oppressive to many, for arms and legs protrude in all directions. In No. 12 ward—the most interesting ward in the hospital because here our imprisoned officers painted their famous war map upon the wall—a poor sergeant lies awake with flushed face and staring eyes gasping through his fourth sleepless night. He had appeared to pass pretty well through an ordinary attack of enteric, and one joyful day he had been "marked" for the Hospital train to the Cape. This meant a chance of being invalided home to England, and he was eager for the start. But bronchitis and pneumonia came on, and with them that form of congested heart said to be connected with the high level at which Pretoria stands—4,500 feet above the sea. Great was his disappointment when he was declared unfit to travel. Then it was that he mentally faced the bitterness of death. Afterwards physical discomfort became supreme. Brandy makes him cough, and milk makes him cough. Bromide will not bring sleep.

The ridiculous town clock of Pretoria struck 12 half an hour ago. It strikes the coming hour at every half-hour. Sister looks at her watch. Just midnight and Christmas Day beginning. She goes out on to the long verandah to look at the sky, and try to feel some Christmas thoughts. The stars are in full radiance. One misses the twinkle they would have on a frosty night such as it probably is at home. And they are not the dear old stars one knows so well. Orion is there, bright as in the north, but just above him shines the Southern Cross, disappointing as ever, a poor substitute for our Great Bear and Pole Star. The Milky Way has a dark, cloudy aspect, far less beautiful than the shimmer it has as seen from England. A bright band of light moves across the sky at intervals. That is the searchlight from the fort above. Boers are at hand in considerable force, and a concerted attack has been talked of as probable. The searchlight is scouring the country all night long, making a concealed approach of the enemy impossible. There is not "Peace upon earth" in South Africa to-night How different the sounds are to night-sounds in England. That unceasing hum is caused by the vibration of innumerable locusts' wings, and an occasional deeper tone is due to the wings of a huge beetle. The cocks never stop crowing, and from time to time a dog barks, and then all the innumerable stray dogs of the town take up the cry and make night hideous.

The twilight is short, and day comes quickly. By 5 A.M. all the men who are fit are out of bed and washing in the compound. Scrubbing and shaving are the order of the day, for Thomas Atkins thoroughly understands making his face to shine. Beds being neatly made and bedsides swept, each by its occupier, the more energetic proceed to pile the table with flowers and pot-plants, and a last legend goes up—a heartfelt one: "God bless all absent friends!" Sister leaves her few sick men and goes to breakfast in a commandeered house said to belong to General Erasmus. Tinned salmon, well iced, and peaches in abundance form a tempting meal at a table covered with roses. Then home she goes to bed at her lodging, past gardens hedged in with honeysuckle, briar-rose, and cypress. It gets hotter and hotter; thunder is about. She wishes with Sidney Smith that she could take off her skin and sleep in her bones.

Unrefreshed, she emerges in the afternoon to find fun fast and furious proceeding in the hospital. The chicken dinner had proved entirely satisfactory, and was followed by a fancy tea, after which all who could collected in the largest ward for a concert. The doctors, sisters, and their friends, were all performing to the best of their ability, among kind friends being a Canadian officer and two from Australia. Piano, harmonium, and mandoline were all pressed into the service, and some roaring choruses testified to the enjoyment of the men. Between the pieces all the talk was of the Boers outside. They had hoped to catch the British off their guard, and feasting, but the garrison was on the alert, and Brother Boer was doomed to disappointment. The last musical performance of the day was by three Highland orderlies, who donned their kilts and played sham bagpipes to everybody's great amusement. None were any the worse for the fun, for those who were sick were in quiet wards undisturbed. Many lay down to sleep with the question on their lips: "Where shall we be next Christmas Day?'

### SWEET CHARITY'S GUIDE TO CHRISTMAS GIVERS.

### CHILDREN'S HOSPITALS—*Continued.*

Hackney Road premises) of the extensive scheme of enlargement upon which the committee of this hospital have entered has at length been commenced and is expected to be completed early in 1903. The total cost of this part of the scheme will be about £30,000. Fifty additional beds, a new operation room, casualty rooms, and other essential accommodation will thus be provided, but much will still remain to be done in the way of building. A nurses' home, an insolation block, and a new out-patient department are all urgently required, but of necessity the erection of these buildings, involving a further expenditure of about £15,000, has been postponed till after the completion of the Hackney Road premises. The committee now have about £13,000 available for building purposes, and most earnestly appeal for the balance of £32,000 required to complete the scheme of enlargement. 18,000 children are treated in the out-patient department, and 760 in the wards annually. Large numbers of cases have to be refused admission for want of room. Help is urgently needed both for the building fund and the general fund. Contributions to the Secretary, Mr. T. Glenton-Kerr.

**Paddington Green Children's Hospital.**—This hospital was established in 1883. It was rebuilt and enlarged in 1895. It provides 46 cots. There is a large out-patient department, where over 1,000 patients are treated every week. The hospital has a Convalescent Home for 12 children at Wealdstone, Harrow. Funds are urgently needed for the maintenance of both the hospital and the home. The hospital owes its bankers £1,500. Secretary, Mr. W. H. Pearce.

**The Hospital for Sick Children,** Great Ormond Street, London, W.C.—The Hospital for Sick Children is the oldest

To only five of the party is that question definitely answered yet. They rest in Pretoria—two orderlies and three patients. They lived and died brave men, and they will never be forgotten by the sister who worked and watched with them that Christmas night.

### CHRISTMAS IN A NEW SOUTH WALES HOSPITAL.

OUR mission, as a convalescent hospital, is to try and make our people forget the ordeals from which they are just recovering, by surrounding them with every comfort and care; with as much brightness and pleasure as possible.

This hospital is over 40 miles away from the city, and far from other habitations, standing in its own grounds of about 500 acres, partly wooded, and partly cultivated. There is a fine lake near the building, and the river winds through the estate. The latter is a favourite resort at all times of the patients, and with its pleasant reaches, shaded by overhanging trees, and grass growing green to the water's edge, makes a splendid lounging place during the hot summer afternoons, which, however, in this dry atmosphere, are seldom unbearable. Indeed, the climate is so delightful, as a rule, under these sunny skies, that we aim at our people living

A Summer Christmas in Southern Climes.

### SWEET CHARITY'S GUIDE TO CHRISTMAS GIVERS.

### CHILDREN'S HOSPITALS—*Concluded.*

and largest children's hospital in the British Empire. £20 a day is required to meet the difference between expenditure and income. Over 2,000 in-patients are treated annually. For 1902 there must be a deficit of at least £6,000. Help is urgently needed. New subscriptions are really required. Debt, £23,500. Secretary, Mr. Adrian Hope.

### HOSPITALS FOR WOMEN.

**Samaritan Free Hospital for Women and Children,** Marylebone Road, N.W.—This hospital is entirely free; it is dependent upon the charitable public for support; it needs £6,000 per annum. Funds wanted at once to meet overstanding liabilities. Committee appeal for bequests and donations towards a new endowment fund. £1,000 will endow and name a bed. £5,000 required to build a new out-door department and to make some enlargements and improvements: these are all imperatively necessary. Secretary, Mr. G. King.

**The Chelsea Hospital for Women,** Fulham Road, S.W.—This Hospital, which has 52 beds, was founded in 1871 for the reception and treatment of respectable poor women and gentlewomen in reduced circumstances. The council appeal for donations and new annual subscriptions (their only reliable source of income) in order to meet increasing demands without incurring a heavy debt, and to pay off a mortgage of £4,000 on the hospital. There is a convalescent home at St. Leonards-on-Sea, which contains 22 beds, but it is not reserved for those only who have passed through the hospital. Secretary, Mr. Herbert H. Jennings.

**The Hospital for Women,** Soho Square, W.—There are 64 beds, of which 25 are closed for want of funds. The

### IN A NEW SOUTH WALES HOSPITAL.

principally in the open air. Christmas comes at the hottest time of the year, and we in the country have neither need nor inclination to hurry over it. To do so would be very undesirable where the thermometer may range at anything from 80° to 110°, and where our people are more or less hors-de-combat. This is the season when doors and windows often stand open all night to catch a cool breeze, and are carefully closed during mid-day hours to exclude the heat—when the lightest clothes are far too weighty, and cooked food is a superfluity. But where we have such ample space and freedom our festivities can be carried out without any of the limitations which abound in a sick hospital, where pleasures must always be tempered by the sight and sounds and needs of acute suffering.

This year we arranged to have our Christmas tree in the open, under the stars. An enterprising young gum tree, with its graceful foliage and perfect shape, was irresistible. It had shot up on the lawn near one of the flower beds and had in consequence been doomed to the axe. Kind friends had, as usual, provided us with the means of giving a present to every inmate, and these, together with ornaments and Chinese lanterns were fastened to the tree by many willing hands on Christmas Eve while the patients were busily engaged inside. Then, when night fell, and we beheld the tree, lighted by its own radiance and Chinese lanterns in the neighbouring trees, surrounded by eager happy faces, the brightly illuminated hospital in the background, it would have been hard to find a prettier picture or a happier crowd at any Christmas festivity. When the last present had been given, the last song sung, and throats could cheer no longer, when tapers had burnt themselves out and bonfires sunk into ashes, when the boys and girls had grown weary of romps and games, we got our excited but happy family to go to bed and ultimately to sleep.

Christmas morning we awoke in a decorated hospital, and to the sound of time-honoured carols. After the previous night's festivities the invitation to "Christians awake, salute the happy morn," was not responded to as promptly as it might have been, but there was a quickening into life when Christmas gifts were brought to light, and merry voices were soon heard exchanging the old greetings. But, oh, how hot it was already. After breakfast only a few felt inclined for a quiet walk in the shade, while others gathered in groups, exchanging reminiscences of Christmas in other lands, under other conditions, and all dwelling lovingly and longingly on "home," wherever that might be for each individual. Then came the dinner, the event of the day, when roast beef, plum pudding, and claret-cup is enjoyed as much in this land of heat and sunshine as in far-away England under less kindly skies. Bonbons, nuts, crackers, etc., enlivened the afternoon for the children, while their elders found a siesta more to their liking. Next came tea-time and more good things, after which a short sacred concert and then to bed.

Boxing Day in the Christmas programme was down for a picnic at the river to everyone's delight, especially that of the children. Early in the morning hampers were packed with requisites for the day's outing, and sent away in carts before the heat of the day. All the halt and weakly people were despatched in good time in charge of a nurse to a shady spot near the river, where the grass was short and green, and where the sight of the water, at least, suggested coolness. There were plenty of sandy banks where the children could paddle in safety if they wished, always with the caution, "Mind the snakes," while swings were quickly put up under the trees, and gaily-covered bags of nuts and sweets were suspended from the trees, feeding the anticipations of the boys.

Soon, some neighbours from a distance joined us, prizes were produced, and races and games became the order of the day, when the last stragglers had come. So the hours flew rapidly. The "grannies" and "grandads" had been made comfortable on rugs, on banks of maidenhair, which grow in profusion, and enjoyed the fun, by proxy, as much as any. A few anxious "Marthas" of both sexes spread the tablecloths, and anxiously watched the water boil for "billy tea," without which no picnic is complete in Australia. Cricket and quoits were patronised by their devotees, while round games were popular with others. So the day passed happily, with intervals for luncheon and tea, accompanied by much pleasant laughter and nonsense, until in the cool of the evening, to the sound of "Auld Lang Syne" and "Home, Sweet Home," everybody wandered back to the lights of the hospital, twinkling through the trees. Strong willing hands help the weak, while the little children are carried, and all are quite ready for rest after their exertions and excitement.

The next day was full of much mysterious whispering and laughing in consequence of the

### SWEET CHARITY'S GUIDE TO CHRISTMAS GIVERS.

### HOSPITALS FOR WOMEN—*Concluded.*

committee very earnestly appeal for additional annual subscriptions and donations. Secretary, Mr. David Cannon.

### MISCELLANEOUS—SPECIAL.

**Cancer Hospital.** Free. Fulham Road, S.W.—Founded in 1851 for the free treatment of the necessitous poor who are afflicted with cancer, tumours, or allied diseases. The nature of the disease makes it necessary to supply both food and dressings in greater quantity and high quality. The annual expenditure amounts to about £12,000, to meet which there are annual subscriptions of £2,000 and dividends £3,400. The balance has to be made up of donations, etc., and generally it means selling out stock to the amount of about £3,000 or £4,000. Secretary Mr. F. W. Howell.

**Dental Hospital of London,** Leicester Square, W.C.—The new hospital is unsurpassed in any other country for the purposes for which it is intended, viz., the alleviation of pain, and the saving of the teeth of the poor, and for the education of our future dentists. Unfortunately, the committee are heavily hampered by debt, unavoidably incurred through being compelled to build on the site they had purchased before sufficient money had been collected for the purpose. The committee had no choice, unless they were prepared to abandon the very suitable site, but to build the hospital, and they now earnestly appeal to the charitable public to help pay off the mortgage of £55,000, and the interest on the loan. Secretary, Mr. J. Francis Pink.

**London Fever Hospital,** Liverpool Road, Islington, N.—The institution is dependent upon voluntary support, and donations and subscriptions will be gratefully received, especially as the alterations and additions to the hospital, now being carried out, and the building of a convalescent preparations for a fancy dress ball to be held in the evening. Many demands were made on matron's and sisters' resources; all sorts of odds and ends were pressed into service, and with much ingenuity and very little expense many striking results were obtained. In the evening the town band came in their neat uniform to make music for the patients, who were transformed into a crowd of strange-looking objects, and as they marched into the gaily-decorated hall were greeted with peals of laughter. Many of the characters were most original, the children being especially grotesque, and their antics provoking shouts of laughter and amusement all the evening. Characters from "Pickwick," from "Uncle Tom's Cabin," and from nursery rhymes were most popular. For the children was provided, in the intervals of dancing, musical chairs, "Jolly Miller," and Hunt the Slipper. After a light and early supper, tired with games and dancing, all were ready to retire, fatigued but happy. So the days went on, each with its own innocent pleasure, until the routine of duty once more settled on the hospital; but surely we prove that "a merry heart is as good as medicine," for it is difficult to believe that many here are recovering from almost fatal ailments, and that the rosy faces of the children when they leave are the same which a short month ago were pale and sharpened by illness. Marvellous cures are wrought by freedom from care, long hours of sleep, wholesome and ample diet, with plenty of innocent pleasures throughout the year, as well as at Christmastide.

### Christmas in a Melbourne Hospital.

This season which brings joy and gladness with it to the homes of so many is not allowed to pass without shedding its benign influence on the hospitals and other charities of Melbourne. A very kindly feeling is shown by the charitable public to make more pleasant for the time the lot of those who have been overtaken by misfortune, and donations in kind, and of many kinds, are sent to make that day, of all days, a little brighter for these poor sufferers.

Not only are abundant creature comforts provided by the authority of the committees of management, but the wards and corridors are decorated with flowers and greenery, many of these being the contributions of visitors and friends of the patients.

I always find that Christmas Eve, or rather the few days immediately preceding Christmas, is a very busy time with all of us, and with me more particularly, from a domestic point of view. The sisters and nurses are decorating the wards, etc.; living as we do in a land of sunshine and flowers, and our Christmas occurring in mid-summer when there is quite a profusion, the effect is very charming to the eye, although we have not the holly, ivy, and mistletoe of Old England at Christmas.

We have 60 beds, and fully one-fourth or more of these are occupied by children, to whom Christmas is a very important season. On Christmas Eve stockings are hung up, and all are in a state of expectation and excitement. I remember one Christmas week going into the laundry and finding two little boys, who had quietly made their way in, busy washing their stockings that they might be clean for "Sandy Claws." One had lighted a fire under the boiler, which he was stoking up with quite a busy air just as I entered, and although I had to cut the proceedings short, I could not help laughing. The incident was so childlike.

Towards midnight on Christmas Eve the stockings are gathered for me by the night nurses, each having on it the name of the child and the number of the ward. These we fill with many surprises, and early in the small hours a nurse replaces each. I also hand to the night nurses the Christmas letters for the patients—which a charitably-disposed gentleman and his wife always provide—as well as one for every member of the staff, accompanied by a handsome card or calendar, so that on waking each patient finds a letter under the pillow.

We are invariably very tired before we can get to bed on Christmas Eve, but when we do, alas! sleep is denied to us. Unfortunately, the building stands close up to the footpath, at the intersection of two streets, one a leading thoroughfare, and as the Christmas bells ring out, above them may be heard the discordant sounds of whistles, concertinas, and the all-too-powerful voices of bands of young Australians who delight in parading the streets on this generally warm summer night until nearly 2 A.M.; then, at last, quietness is restored and we get a chance to sleep.

Christmas morning is heralded in by the sounds of blasts of toy trumpets and bugles issuing from the various wards, as the little ones have been to the

### SWEET CHARITY'S GUIDE TO CHRISTMAS GIVERS.

### MISCELLANEOUS—SPECIAL—*Continued.*

home, are taxing to the utmost the resources of the institution. Secretary, Major W. Christie.

**London Lock Hospital and Rescue Home,** Harrow Road, W.—Help is required for this valuable institution. There has been a continual flow of patients to the Hospital, some of whom have found their way to the home, in several cases with very good results. It is the desire of all connected with the Hospital and Home to do the utmost in their power to succour those who are in want of a helping hand. Secretary, Mr. A. W. Cruickshank.

**Royal London Ophthalmic Hospital,** City Road, E.C.—This hospital, like all hospitals, has been hard hit during the past year owing to the other claims pressed on the charitable public. But its position is different from most other hospitals, because it has not a single penny of invested funds which can be realised. The committee are most reluctant to close a hospital which has been working for nearly one hundred years, which has a world-wide reputation, and which relieves about 40,000 patients annually. The new building in City Road is perfectly designed and equipped for its special object, and worthy of the work and the teaching of the oldest and largest Eye Hospital in Great Britain. On these grounds the committee appeal for help, and beg that contributions may be sent in time to save such a public calamity as the closing of this great institution. Secretary, Mr. Robert J. Bland.

**Royal Orthopædic Hospital,** 287 Oxford Street, W.—The falling off of charitable support which, owing to the effects of the prolonged war, is now being so much felt, has greatly interfered with the work of this hospital. Notwithstanding the closing of two wards, thus reducing by 10

### IN A MELBOURNE HOSPITAL.

bottom of their beds and found their stockings, with which they are so busy that the nurses find it hard to get them to take their breakfasts.

About 11 A.M. on Christmas Day all the children who are able to be up, as most of ours are, are brought down to my room or to the office and there they choose according to sex, and to the sisters' ideas of their various tastes, a really handsome toy or gift, most thoughtfully provided by a gentleman for each child in every institution here every Christmas. There are also other little parcels of lollies, etc., which we provide by way of supplement, and the pleasure of these new possessions and the comparisons made by each, fills in the time well until the dinner hour.

Despite the difference in the climate at this season of the year the old English custom of roast beef and plum pudding for dinner is observed throughout Australia, and although the temperature may be up in the nineties all seem to enjoy it. The ward tables are laid by the nurses, and very nice they look with plenty of vases of flowers and tall grasses and plates of bananas, pineapples, apricots, and peaches—all fruits are plentiful and cheap here just then—whilst jugs of iced raspberry drinks, lemonade, gingerbeer, and English bottled ale, are provided as beverages to suit the different tastes. For the very old people in the seventies, eighties, and even nineties (principally cataract cases), jugs of whisky punch are provided. One of our honoraries provides tobacco for the male patients and all smokers that are up retire to the smoking shed and enjoy a pipe after dinner.

Visiting is generally allowed on Christmas afternoon, and many small presents are brought in by the friends to each little patient who watches eagerly for their appearance. The friends on their part seem to prize the privilege of seeing the little absentees from the home circle, the Australians being particularly attached to their children, and Christmas being very much observed by them.

After the visitors go, tea time comes round and all enjoy the excellent Christmas cake provided by the hospital. When the day is over many are the expressions of satisfaction from young and old, with regard to the Happy Christmas they have spent in the Victorian Eye and Ear Hospital.

### CHRISTMAS IN A HOSPITAL IN THE AUSTRALIAN GOLDFIELDS.

In trying to give some idea of how we pass our Christmas here, the first and most important point to realise is that the season is spent amidst the trying heat of a tropical summer, which in Bendigo is at times overpowering. Notwithstanding this drawback, we do our best to make those who are compelled, through sickness, to be absent from their homes at such a time as happy as we possibly can. But it is no uncommon thing for the thermometer to register 108 in the shade on Christmas Day.

The Bendigo Hospital is a most picturesque building, standing in 10 acres of land, laid out with numerous pathways, and thickly-studded trees and shrubs of every description, which afford plenty of shade for the convalescent patients. The main drive sweeps majestically from the entrance gates to the front entrance of the main hall. On the right of the drive stands the nurses' home, built by public subscription to commemorate the Jubilee of our late beloved Queen. It is a triumph of modern architecture, and would compare favourably with similar homes attached to the colossal institutions in the old world, and it is furnished throughout with the latest ideas of cosiness and comfort in every detail. The rooms are carpeted with Brussels carpet and all the best of modern furniture has been provided. In the nurses' sitting-room there is a magnificent piano which cost £85, purchased by the special efforts of the nurses and matron, and I am pleased to state that at present the balance owing is only £4. The hospital contains 160 beds with five wards which can accommodate 30 patients each. We have also a set of children's wards, the medical accommodating 16 and the surgical 11. Detached, a good distance from the main buildings, are lunacy wards capable of taking about 10 patients of both sexes; there is also a contagious cottage of similar capacity.

At Christmas the adult patients are generally visited by their relations, who on Christmas Day bring to those who are allowed their Christmas dinner. I am sorry to say that these are only the fortunate few. The other patients are specially seen to by the matron and nurses. The wards are all decorated with pot plants, and we have plenty of flowers from our flower garden in the grounds.

As regards the little ones under our care we try in

### SWEET CHARITY'S GUIDE TO CHRISTMAS GIVERS.

### MISCELLANEOUS—SPECIAL—*Continued.*

the available number of beds, a heavy debt has accrued during the past 12 months. At least £1,000 is required to meet all the claims due to Michaelmas, and unless generous help is given during the next few weeks, a heavy liability will be carried forward to the new year. Secretary, Mr. Tate S. Mansford.

**St. Luke's Hospital,** Old Street, E.C. — This well-known institution has been treating mental diseases for the past 150 years. Is now solely devoted to our great "middle-class" population whose means are limited. Two hundred beds are nearly always in use, and over 25,000 patients have been treated since the opening. The convalescent home in connection with the hospital is at St. Lawrence-on-Sea. Extensive improvements to increase the comfort of the patients have this year been completed, and the committee have this year spent £4,000 in order to acquire the freehold of the convalescent home. Funds are very urgently needed. Secretary, Mr. W. H. Baird.

**Surgical Aid Society,** Salisbury Square, E.C. The net expenditure for the year amounted to £15,374, of which 82 per cent. was for actual relief. During the past year the large total of 27,887 appliances were given away. There is ample scope for very considerable extension of these benefits, and, therefore, the committee earnestly appeal for contributions. Secretary, Mr. Richard C. Tresidder.

**The Royal Sea-Bathing Hospital.**—A national institution, founded at Margate in 1791, for the special treatment of surgical tuberculosis. Diseases of the hip, spine, bones, joints, glands, etc., are amenable to treatment here. Limbs, apparently in a hopeless condition, demanding amputation

**IN THE AUSTRALIAN GOLDFIELDS.**

the same way to instil a little happiness into their lot. The wards are decorated in a similar manner as the adults', and we have that hoary friend, Santa Claus, to visit them. Our plan is carried out in the old home style, and many of the children's friends and others bring ample supplies of toys and sweets on Christmas Eve. During the silence of the night the sisters and nurses accompany Santa Claus and help him to distribute his wares. Each little boy and girl is provided with the proverbial stocking to hang at the usual place. The nurses creep stealthily in and out of the wards in fear and trembling, lest some wee one should be sleeping with one eye open and give poor nurse away in the morning. The new-born day is young indeed when the tiny feet are off to explore the contents of their several stockings, and the shrieks of childish delight tell of the surprises which Santa Claus has left in his wake. In fact, the nurses are more than repaid, both by the delight of the children and by the pleasure afforded to the grown-up patients for their labour of love at the Christmas season.

### CHRISTMAS IN A TORONTO HOSPITAL.

In the usually still corridors of the hospital there is an air of excitement, which communicates itself even to the casual visitor. Mounds of holly and rolls of wreathing lie here and there on the floor in a state of confusion which, at any other time, would horrify the head nurse; but to-day she appears blissfully unconscious of the disorder and smiles approvingly as one or other particularly artistic form of decoration is added to the hall. Mounted on ladders are the available "men"—some orderlies, some doctors—engaged in the common task of making the place "look like Christmas." It is Christmas Eve. In the wards the nurses who can be spared from the ordinary duties are busy, hanging holly over the lights, winding wreathing around pillars, and, in response perhaps to an eager request from some poor fellow with a memory of happy Christmas times long ago, placing a piece of mistletoe at the head of the bed. Now and then a patient begs for a sprig of the glistening holly, with its ripe-red berries, and is happy for the rest of the evening with this foretaste of Christmas.

And now the tea-bell sounds through the house, and the workers hasten to put the finishing touches before descending to the dining room. What a meal that Christmas Eve tea is! In keeping with the time-honoured custom, mince pie is served in plenty, and is done ample justice to by the hungry gathering. For decorating is real work, though pleasant, and therefore an appetiser; but the sense of weariness in this case is very satisfactory, fraught as it is with the thought that the labour has helped to make the sufferers above-stairs feel that though they are to spend Christmas in bed, yet it will be Christmas.

Orders have been issued that all decorating work must be finished before tea so that the patients may not be disturbed later in the evening. Accordingly, the nurses complete the evening toilets, make the reports to the night nurse, and retire to their rooms, where mysterious-looking parcels await them. By common consent none are opened on Christmas Eve, but of course "guessing" is quite in order, and it is indulged in to an unlimited extent. However, half-past nine arrives, the lights are out, and over the whole house falls the stillness of night. In the wards the nurses in charge step softly to and fro, performing their duties this night as perhaps on no other night in the year; always gentle in their treatment of patients, they now show a special tenderness. From a wee patient's childish lips, relaxed in sleep from their tension of suffering, comes the murmur of "Santa Claus," and the nurse bends over the little form, gently covering the bare arms thrown apart, and whispers, as she places by the pillow a doll dressed in the hospital uniform, "Poor little darling! I'll be your Santa Claus."

The hours of the night pass on, midnight comes with its chimes, telling the old old story of the blessed Christmastide; five o'clock, and there is a stir in the nurses' quarters, a general awakening, exchanges of Christmas greetings, a hurried but rapturous survey of gifts, and everyone is dressing as quickly as possible, in order to get to the board room to awaken the patients with the sound of Christmas carols. The night nurses have been duly cautioned to soothe to sleep again any early waker, that the first knowledge of Christmas morning may be the sweet air of "Hark! the Herald Angels sing."

And now the board room doors are opened wide; the staff, nurses and servants assembled, and from the piano rings out the well-known air. As the

### SWEET CHARITY'S GUIDE TO CHRISTMAS GIVERS.

### MISCELLANEOUS—SPECIAL—*Concluded.*

recover here. Two-thirds of the patients come from London. The treatment is (1) surgical; (2) sea-bathing in summer and hot sea-water baths in winter; (3) good nursing and abundant food; (4) open air; (5) medicine. Owing to failure of legacies, £3,000 is required to prevent the closure of beds. Secretary, Mr. A. Nash, 30 Charing Cross, S.W.

### GENERAL CHARITIES.

**Association for the Oral Instruction of the Deaf and Dumb.**—The first association to publicly introduce into the United Kingdom the German or pure oral system for teaching deaf and so-called dumb children to speak *viva voce*, and to understand the spoken words of others by lip-reading. The expenses which are very heavy, are met by voluntary contributions and fees, which fall short of what is needed by about £500 per annum. Director, Mr. Van Praagh, 11 Fitzroy Square, W.

**City of London Truss Society,** 35 Finsbury Square, E.C.—At the present time about 10,000 of both sexes and all ages are treated annually. The premises have recently been enlarged to provide for the increased number of patients, and additional funds are greatly needed to meet the growth in the expenditure. Over 565,900 patients have already been relieved. Secretary, Mr. John Whittington.

**Irish Distressed Ladies Fund,** 411 Oxford Street, W.—Relief is given independently of any question of politics or religion; employment is found, when possible, for those able to work; pecuniary help is given to the aged and infirm; and the education of children is paid for. Secretary, General W. M. Lees.

### IN A TORONTO HOSPITAL.

strains rise through the corridors, the patients awake to hear the sacred story of the Incarnation. Gradually it is taken up in the wards, the weak, quavering notes following the lead of the fresh young voices below, and now the whole house is uplifting the hymn of praise to "God of God, Light of Light, very God and very Man."

The music ceases, and with the Christmas message "Peace on earth, goodwill to men" speaking in every heart, there is a more general interchange of good wishes.

When the nurses take their posts "on duty," they find the morning routine almost impossible. There are parcels to be distributed to the patients, many of them to be opened, where poor rheumatic hands refuse to untie the string; presents to be admired, and eulogies on the givers to be listened to and endorsed. Breakfast is served amidst the excitement, but is scarcely appreciated. There are a few, however, too ill even to notice what Santa Claus has done for them, and these are attended with loving care and skilful hands.

Outside the keen frosty air is alive with the sound of church bells. There has been a heavy fall of snow during the night, and the pure white garment has clothed Mother Earth to a depth of two feet or more. The bright Canadian sunshine glistens on the snow-clad boughs of the trees, wherein the birds, infected with the music of the bells, pour forth rich strains of joy. Swiftly over the snow speed the cutters; the horses' bodies encircled by sleigh bells which seem also to sing a joyous song. One after another the visiting doctors drive up to the door, and enter the institution with a "Merry Christmas" on their lips and in their eyes. This is the office boy's harvest, for many a silver piece finds its way from the doctors' pockets to his hand. As they pass through the wards they bring with them a fresh touch of the bright morning outside, and their cheery greetings to the patients make a pleasant feature in the morning's proceedings.

In due time the Christmas dinner is served, first to the patients, afterwards to the nurses and staff. To those who are able to partake of them, turkey and plum pudding are given, and many poor creatures who could not afford these luxuries at home enjoy a real Christmas dinner. In each piece of pudding is a small sprig of holly, making a bright show on the tray.

At the nurses' table the fun is fast and furious, and turkey disappears with marvellous rapidity, only to be followed by a generous supply of plum pudding. With the dessert, memories of home customs are revived and in turn given a trial, ending in huge success or laughable failure, received with equal good humour and enjoyment. It is, perhaps, a little hard to return to the wards and perform the ordinary duties, when the whole world is having a holiday, but the sight of the friends gathered around the beds of their charges, the happy smiles as gifts are presented and Christmas kisses given, drive away the momentary regret, and they soon have the rooms in order, ready for the visit of the choir boys. For this is one of the time-honoured customs at Grace Hospital—the Christmas afternoon visit of choir boys from a neighbouring Episcopal Church. The custom was originated by a kind-hearted clergyman, in his desire to do his share in making the patients' Christmas a happy one. It has been perpetuated by his successors in Toronto. From ward to ward the youthful choristers pass, singing the familiar hymns, assisted generally by patients and nurses. Thus, not only are the patients cheered and comforted, but these young soldiers, full of strength and vigour, learn the lesson of sympathy for the wounded ones in the hard battle of life.

The visitors' bell rings and adieus are said. After tea the weary patients are quite satisfied to have the beds fixed for the night and the lights turned low. A happy expression of content is in their eyes as the nurses bid them good night, and soon after they are dreaming of angel voices singing, ever singing, the wondrous song of peace.

### CHRISTMAS IN A FIJI HOSPITAL.

It may be difficult for the untravelled reader to picture a bright, sunny, hot Christmas Day, every tree wearing the greenness of summer, yet lacking none of the freshness of spring; every door and window thrown wide open, the nurses dressed in uniform of lightest zephyr, and the bare-footed brown-skinned wardsmen flitting noiselessly about their duties with turbaned head and graceful loin-cloth.

Such are, however, our normal circumstances in the Southern hemisphere, at the hospital from which

### SWEET CHARITY'S GUIDE TO CHRISTMAS GIVERS.

### GENERAL CHARITIES—*Continued.*

**London Orphan Asylum**, Watford.—A special appeal is made by the manager for a sum of £4,000. Owing to gaps caused by the death of some old friends and changes in the means of others, as well as the demands made by "special" national appeals, the list of the annual, as well as occasional, contributors has been seriously impaired, and new friends are earnestly asked to support the cause of the widows and the fatherless. The charity receives from all classes and localities those little ones who are not eligible for admission to trade and membership institutions. Since it was founded, upwards of 80 years ago, it has equipped for the battle of life 6,300 destitute fatherless boys and girls of respectable descent. Secretary, Mr. Henry C. Armiger, at the office, 21 Great St. Helens, E.C.

**London Schools Dinner Association.**—In view of the attempts which are made to throw upon the rates burdens which charity is competent to bear, attention is especially directed to the work of this association. It makes grants to provide cheap or free meals for necessitous children attending the public elementary schools of London, and is doing an excellent work. The annual income now averages about £1,500. Further subscriptions are urgently needed and will be gratefully received to enable the association to be in a position to make immediate grants in cases of proved necessity recommended by responsible local committees. Hon. Treasurer, Lord Kinnaird. Secretary, Mr. T. A. Spalding, 117 School Board Offices, Victoria Embankment.

**Mary Wardell Convalescent Home for Scarlet Fever,** Stanmore, Middlesex.—This being the only home for convalescents from scarlet fever, the demand on its 40 beds is great. The committee have been compelled by the District Council to reconstruct the drainage system of the home in order to connect it with the public sewer which has recently

### IN A FIJI HOSPITAL.

I write. In our latitude, at the Yule season, the first streaks of dawn begin to spread over the horizon soon after four o'clock, and by five the morning is quite light. But the hours before and just after sunrise are moderately fresh and cool, and the night nurses even find comfort in a wrap, for they sit, when not actually engaged with a patient, in the spacious ward verandahs quite freely exposed to the refreshing night air, thanks to the fewness of mosquitos and their entire freedom from the malarial parasite. From nine o'clock the day is usually very hot, until sunset. Mistletoe and holly are wanting here, and a stray leaf or so of either of these emblems, sent, it may be, from the snow-clad Motherland by thoughtful friends, finds sympathetic favour when it reaches us with the home mails, some thirty or forty days after being posted. But of ferns and palm leaves, bamboos and gay foliaged shrubbery, we have galore, while the many white or yellow flowers afford capital material for decorative purposes. Christmas in the natives' wards, however, is not a very special festival, for all the Christian patients who are able like to go home to their villages to spend the holiday season, leaving only those who, by reason of their ailments, nationality, or creed, care but little about our time of rejoicing.

Loremongers have it that Christmas festivi tie

Corridor of a Ceylon Hospital.

### SWEET CHARITY'S GUIDE TO CHRISTMAS GIVERS.

### GENERAL CHARITIES—*Continued.*

been laid down, and funds are urgently wanted to meet this enforced expense. An appeal for £1,000 is made to pay off this and other debts resting on the home. Donations to Miss Mary Wardell.

**Orphan Working School**, Haverstock Hill, N.W., and Hornsey Rise, N. Offices, 73 Cheapside, E.C.—A national and undenominational institution which maintains 500 children, varying in age from infancy to fourteen or (in special cases) fifteen years. It is greatly in want of funds at the present time. Appeal is made specially for new annual subscriptions, the income of the charity being more than £2,000 below what is needed to meet the expenditure. Secretary, Mr. Alexander Grant.

**Our Sailors—Royal Alfred Aged Merchant Seamen's Institution** (established 1867) is the *only* institution which gives—irrespective of rank, ports of service, or place of abode—a home or a pension to the British merchant sailor when old and destitute. The annual subscriptions do not reach £4,000, and the disbursements (including out-pensions and total support of inmates) are nearly £9,000. From want of funds the committee are compelled to keep in painful suspense hundreds of poor aged applicants. Secretary, Mr. J. Bailey Walker; office, 58 Fenchurch Street, E.C,

**Royal Albert Orphan Asylum**, Bagshot, Surrey.—This charity requires about £3,000 to meet the outstanding liabilities of the current year. In spite of the difficulty which the committee experience in raising funds, they are loth to curtail the good work, and still maintain the full number of

### IN A FIJI HOSPITAL.

date from very ancient times, and were customary in Northern Europe before the Christian era, that they represent, in fact, a survival of those heathen institutions which were adopted by sun-worshipping primitive man for the purpose of marking the passage of the seasons. Be that as it may, our natives, who were never sun worshippers, and who have no original method of marking eras or dates, were equally lacking in any prototype of Christmastide, and they set no great store by our Church festival in the present day.

Nevertheless, in Fiji, we try to make the day as happy as possible for those who are with us, and the exuberance of evergreen plants makes the task of decorating an easy one. After all, it is only the difference between having the greenery outside the buildings or inside them; it is always in view from the verandahs all the year round, and the patients, if so minded, can lie and feast their eyes on the ever-changing hues of forest, reef, and sky without rising from their mats.

But it is the digestive, rather than the visual, organs which may be truthfully termed the portals to a native's sense of satisfaction. His æstheticism, if so it may be called, is of the physical rather than of the psychical order, and, in these Southern seas, the *summum bonum* of all his Christmas ideals may be stated in the one word "vuaka" (pork). In this it appears he is not singular, for the mighty men of Troy and the ancient chiefs of Polynesia were equally famous for their banqueting proclivities, and pork was a diet no less admired by the Greeks of old than it is by the South Sea Islander of this day. Give the latter his fill of it, with a sufficiency of vegetable concomitants to render the feast duly appetising, and every other consideration for his happiness may be cast into oblivion, without risk of sacrificing any fraction of his regard for you. No doubt it is a far cry from the barbaric brown man who less than half a century ago was still enjoying his predilection for cooked members of his own species—to the polished man of letters. Yet no less brilliant an essayist than Charles Lamb has expressed his admiration for the succulent flesh of swine. And that in no half-hearted terms, for in his amusing "Dissertation upon Roast Pig," he maintains that "of all the delicacies, roast pig is the most delicate." Perhaps the very predilection I have mentioned in the case of the savage has engendered, or at least intensified, his modern liking for baked pork; it is a fact that in the islands of these seas, the native still speaks jocularly of his father's cannibal *pièce de résistance* as "vuaka balavu," or long pig. Certain it is that Christmas affords him an opportunity for imitating, savages like, the feasting proclivities of his betters which culminate at that season; one, too, which he does not fail to turn to account.

This kind of satiety would obviously be out of place in a hospital ward, and could only be permitted if promptly followed by unwelcome libations from the dispensary. Hence our Christmas arrangements are quite simple and commonplace, our European patients being usually few in number, and either lazily indifferent to the ethics of the season, or too ill to pay attention to them.

A few of our patients are negritos from the Solomon Islands. They are of a receptive nature, and like to ape the customs of white men. Accordingly they add to the orthodox pig a pudding, and portions of it are duly presented to the sister for issue to such of their countrymen in hospital as may be permitted to partake. The pudding usually consists of harmless vegetables and fruit combined, such as *taro*, bananas, sugar-cane juice, and grated cocoanut. One of the Christmas puddings that I saw was 30 feet in length, and on another occasion the procession of men, each bearing a pig, baked whole, on his shoulder, numbered 81. Europeans invariably eschew native pork on account of its parasites and the abominable habits of the animal, but I can assure nurses in more civilised lands that there are few Christmas dinners more charming than a turkey well dressed in a native oven or *lovo*, with a few freshly-gathered chillies inside it, and served up with bread-fruit, or a nice *taro*, provided you can save the gravy.

It is as well, however, to slay a bush hen in time to stew down in case of accident, for though the turkey bakes lusciously in its own tasty juices, it is no easy matter to secure these from escape through rents in the leafy capsule during the process of its disinterment from the *lovo*.

From a strictly nursing point of view, the few weeks following Christmas-tide are of more importance than the season itself, for we have generally a considerable increase in the number of patients in consequence of the indiscretions for which the festival affords temptation, the natives regarding it as a special opportunity for dietetic license.

### SWEET CHARITY'S GUIDE TO CHRISTMAS GIVERS.

### GENERAL CHARITIES—*Concluded.*

inmates. A very novel and interesting scheme is being issued to enable children in better circumstances to aid by small contributions the 200 orphan boys aud girls in the asylum. Full information will be given by the secretary and superintendent, Mr. H. W. Tatum, 62 King William Street E.C.

**Royal Association in Aid of the Deaf and Dumb**, Oxford Street, W.—The objects of this society are to visit deaf and dumb persons at their own homes, and to assist them in sickness and distress. During the past year 3,583 visits were paid to the deaf and dumb, 1,002 visits were made to employers and others on their behalf, 195 were relieved, and 65 provided with work and apprenticed. An increased reliable income is needed to maintain and extend the present work. Secretary, Mr. Thomas Cole.

**Royal Blind Pension Society,** 237 Southwark Bridge Road, S.E.—This society provides pensions, by monthly instalments, for 1,070 blind people. Further contributions, particularly annual subscriptions, are required to enable it to give aid to 200 eligible candidates who are seeking the society's help. Secretary, Mr. W. E. Terry.

**Royal Society for the Prevention of Cruelty to Animals,** Founded 1824.—The labour of other charities is divided among many associations, but this charity stands alone—the defender of defenceless dumb animals. In 1900 this society obtained 7,894 convictions against cruel persons in different parts of the country. These proceedings involved great cost to the society, and an earnest appeal is made for increased financial support to prevent a curtailment of operations. Secretary, Mr. John Colam, 105 Jermyn Street, S.W.

## Figures and Tables

**Figure f1:**
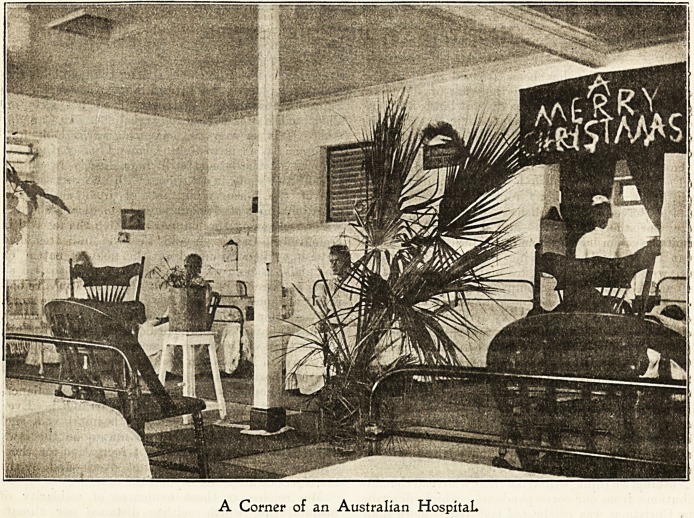


**Figure f2:**
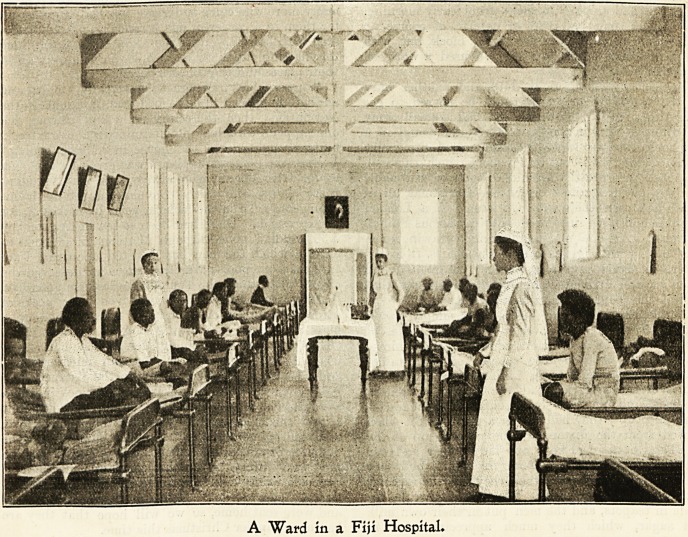


**Figure f3:**
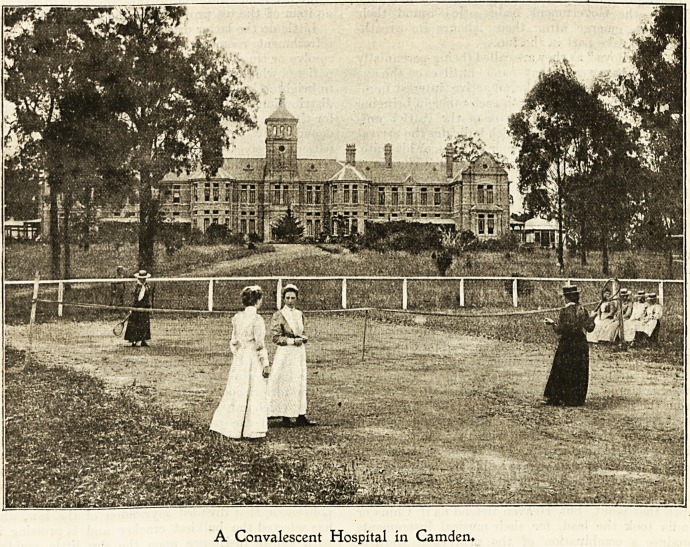


**Figure f4:**
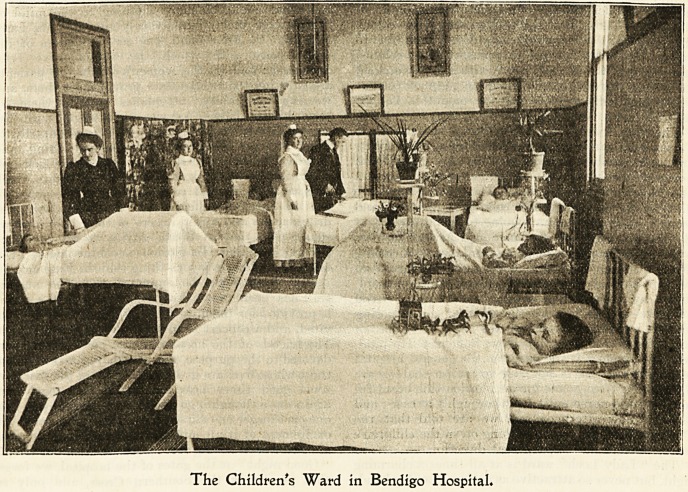


**Figure f5:**
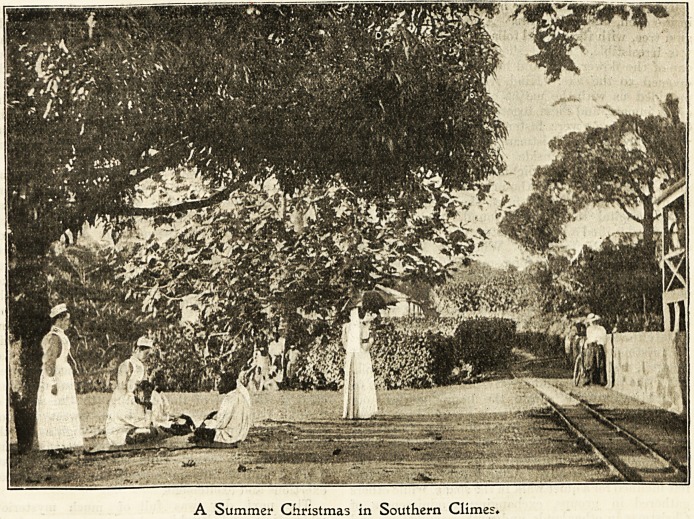


**Figure f6:**